# A retrospective study on Chagas disease detection and management in Tremedal and Novo Horizonte: Insights prior to the Oxente Chagas Bahia Project

**DOI:** 10.1371/journal.pntd.0014569

**Published:** 2026-07-24

**Authors:** Tycha Bianca Sabaini Pavan, Anderson Luiz Pimentel Ferreira, Daniel Dias Sampaio, Lívia Dórea Dantas Fernandes, Carlos Eduardo Lins Franca Piau, Larissa de Carvalho Medrado Vasconcelos, Isadora Cristina de Siqueira, Fred Luciano Neves Santos

**Affiliations:** 1 Advanced Public Health Laboratory, Gonçalo Moniz Institute, Oswaldo Cruz Foundation (Fiocruz-BA), Salvador, Bahia, Brazil; 2 Interdisciplinary Research Group in Biotechnology and Epidemiology of Infectious Diseases (GRUPIBE), Gonçalo Moniz Institute, Oswaldo Cruz Foundation, Salvador, Bahia, Brazil; 3 University Hospital Professor Edgard Santos, Salvador, Bahia, Brazil; 4 General Hospital Roberto Santos, Salvador, Bahia, Brazil; 5 Federal University of Western Bahia, Barreiras, Bahia, Brazil; 6 Maurício de Nassau University Center, Barreiras, Bahia, Brazil; 7 Eurico Dutra Municipal Hospital, Barreiras, Bahia, Brazil; 8 Laboratory of Investigation in Global Health and Neglected Diseases, Gonçalo Moniz Institute, Oswaldo Cruz Foundation, Salvador, Bahia, Brazil; 9 Integrated Translational Program in Chagas Disease from Fiocruz (Fio-Chagas), Oswaldo Cruz Foundation (Fiocruz-RJ), Rio de Janeiro, Rio de Janeiro, Brazil; Centro de Pesquisa Gonçalo Moniz-FIOCRUZ/BA, BRAZIL

## Abstract

Chagas disease (CD) remains a persistent public health challenge in endemic areas of Brazil, where access to diagnosis, clinical staging, and etiological treatment is often limited. Tremedal and Novo Horizonte, municipalities in the state of Bahia, continue to report chronic cases despite advances in vector control. This study provides a retrospective assessment of CD detection and clinical management in these municipalities prior to the full implementation of the Oxente Chagas Bahia Project. We analyzed individuals tested for CD serology between 2019 and 2023 using data from the Laboratory Environment Manager (GAL) system and electronic primary care records (e-SUS). Sociodemographic characteristics, seropositivity, turnaround time for serological testing, and clinical manifestations were evaluated. A total of 472 individuals underwent serological testing. Testing volume increased markedly in Tremedal over time, whereas Novo Horizonte initiated screening only in 2023. Fourteen individuals (3.0%) tested positive for anti-*Trypanosoma cruzi* antibodies, all from Tremedal; 13 were included in the clinical analysis. The median age of confirmed cases was 52 years, and 61.5% were female. Among the 13 confirmed cases, the indeterminate form predominated (61.5%), followed by the cardiac and mixed forms (15.4% each) and the digestive form (7.7%) Electrocardiographic conduction abnormalities predominated among individuals with cardiac involvement, while severe digestive manifestations, including megacolon and megaesophagus, were documented in three individuals. Median turnaround time for serological results was longer in Tremedal than in Novo Horizonte. Documentation of etiological treatment with benznidazole was limited, with treatment records identified for only two of the thirteen confirmed cases in the available medical records. These findings highlight the ongoing burden of chronic CD, substantial gaps in screening and treatment, and the need to strengthen diagnostic access and guideline-based management. This study establishes a critical pre-intervention baseline for evaluating the impact of the Oxente Chagas Bahia Project in endemic municipalities.

## Author summary

Chagas disease (CD) is a neglected tropical disease that mainly affects people living in socially vulnerable and rural areas of Latin America. Although important advances in vector control have reduced transmission in Brazil, many individuals infected decades ago remain undiagnosed and develop chronic heart or digestive complications due to delayed access to healthcare. In this study, we analyzed how CD was detected and managed in two endemic municipalities in the state of Bahia, Brazil, before the implementation of a public health initiative known as the Oxente Chagas Bahia Project, which aims to strengthen CD surveillance, evaluate the performance of rapid diagnostic tests under real-world primary care conditions, and expand access to clinical evaluation and etiological treatment within the Brazilian Unified Health System. By reviewing laboratory records and primary healthcare data from 2019 to 2023, we found that access to diagnostic testing differed substantially between municipalities and that confirmed cases were identified only where screening was more actively performed. Among individuals diagnosed with CD, many already presented cardiac or digestive involvement, and few had documented access to antiparasitic treatment. Our findings reveal important gaps in screening, diagnosis, and clinical management at the primary care level and suggest that the absence of detected cases in some settings likely reflects limited testing rather than absence of disease. This study provides a baseline picture of CD burden and healthcare gaps in endemic rural areas and highlights the need to expand systematic screening, improve diagnostic efficiency, and strengthen access to appropriate clinical care.

## Introduction

Chagas disease (CD) is a neglected, anthropozoonosis caused by the protozoan parasite *Trypanosoma cruzi*, first described in the early 20th century [1]. Despite major advances in vector control and blood-borne transmission screening, CD remains a substantial public health challenge in Latin America [2] and disproportionately affects socially and economically vulnerable populations [3]. In Brazil, the persistence of CD reflects not only historical exposure but also structural barriers to timely diagnosis, access to care, and longitudinal clinical follow-up, particularly in rural and resource-limited settings [4–6]. These barriers contribute to underdiagnosis, delayed treatment, and the progression to advanced cardiac and gastrointestinal forms of the disease.

Transmission of *T. cruzi* occurs predominantly through hematophagous triatomine insects (kissing bugs); however, congenital, oral, transfusion- and transplant-related, and laboratory-acquired routes continue to play a relevant epidemiological role [7]. Independent of the route of infection, CD typically begins with an acute phase that is often asymptomatic or oligosymptomatic. In untreated individuals, the infection may progress to a chronic phase marked by low or intermittent parasitemia and progressive tissue damage. Although 60–70% of chronically infected individuals remain in the indeterminate form, approximately one-third develop clinically significant manifestations, most commonly Chagas cardiomyopathy and, less commonly, digestive involvement such as megaesophagus or megacolon [8]. Chagas cardiomyopathy arises from chronic myocardial inflammation, progressive fibrosis, autonomic dysfunction, and microvascular disease driven by persistent parasite-induced and immune-mediated injury, leading to conduction abnormalities, ventricular dysfunction, arrhythmias, and heart failure. Digestive manifestations arise from progressive destruction of the enteric nervous system caused by chronic *T. cruzi* infection, leading to severe motility disorders of the esophagus and colon. Both forms of chronic CD lead to substantial morbidity, reduced quality of life, and increased mortality, underscoring the need for early diagnosis and continuous clinical monitoring [9–11].

In Brazil, the elimination of *Triatoma infestans* as a domiciliary vector in 2006 represented a milestone in vector control [12]. Nevertheless, native triatomine species capable of sustaining domestic and peridomestic transmission cycles persist in several regions, including the state of Bahia. Tremedal and Novo Horizonte remain historically recognized endemic municipalities where residual transmission and the cumulative burden of chronic CD continue to occur. Although *T. infestans* has not been detected in these municipalities since 2011 and 2015, respectively, entomological surveys have documented the continued capture of secondary triatomine species in both settings. In Tremedal, species identified include *Triatoma sordida*, *T. pseudomaculata*, *Panstrongylus geniculatus*, and *P. lutzi*, with *T. cruzi* infection confirmed in specimens of *T. sordida* and *P. geniculatus* [13]. In Novo Horizonte, *T. sordida*, *T. lenti*, *T. pseudomaculata*, *T. melanocephala*, *T. lutzi*, *P. diasi*, and *P. geniculatus* have been captured, with *T. cruzi* infection confirmed in a specimen of *T. pseudomaculata* [14]. The recurrent capture of infected secondary species indicates an ongoing risk of vectorial transmission in both municipalities.

These municipalities constitute key sites of the Oxente Chagas Bahia Project [15], an initiative designed to address critical gaps in CD control through strengthened surveillance, validation of rapid diagnostic tests under real-world conditions, and expanded access to clinical evaluation and treatment within the Brazilian Unified Health System (SUS). The integration of epidemiological, clinical, and health-system components within this framework enables a reassessment of the baseline status of CD in endemic municipalities prior to large-scale interventions. This baseline is necessary to accurately interpret the effects of diagnostic innovations and to identify structural and clinical limitations in disease management.

This retrospective analysis describes clinical management of CD in Tremedal and Novo Horizonte between 2019 and 2023, prior to full implementation of the Oxente Chagas Bahia Project. Specifically, we review sociodemographic features of the municipalities, access to CD diagnosis, CD testing turnaround time, and the spectrum of chronic cardiac and gastrointestinal disease in individuals with confirmed CD in these regions. This pre-intervention characterization seeks to guide targeted clinical strategies, strengthen surveillance, and support evidence-based public health decision-making in endemic settings.

## Methods

### Ethics statement

This study received approval from the Institutional Review Board for Human Research at the Gonçalo Moniz Institute, Oswaldo Cruz Foundation (IRB/IGM/Fiocruz-BA) Salvador, Bahia, Brazil, under protocol number 70324323.0.0000.0040, approved on 28 July 2023. All procedures complied with national and international ethical standards for research involving human participants. Written informed consent was obtained from all individuals included in the clinical assessment, except for two cases. One individual died years before the time of data collection, and in the case of the pediatric participant, the family moved to another state and could not be contacted despite multiple attempts. In these two situations, the IRB waived the requirement for written informed consent.

### Study area

**Fig 1:**
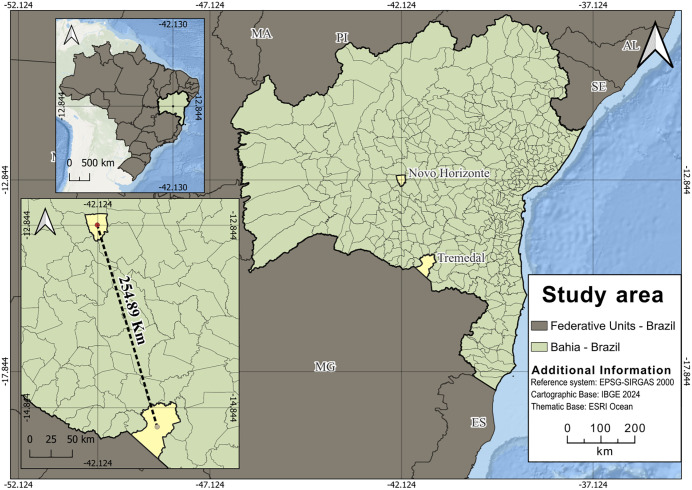
Map of Bahia showing the study area. A public domain digital map was obtained from the IBGE (https://www.ibge.gov.br/geociencias/organizacao-do-territorio/malhas-territoriais/15774-malhas.html) cartographic database in shapefile format (.shp) and then reformatted and analyzed using QGIS version 3.22.16 (Geographic Information System, Open-Source Geospatial Foundation Project: http://qgis.org). IBGE: Brazilian Institute of Geography and Statistics.

The study took place in the municipalities of Tremedal and Novo Horizonte, in the state of Bahia, northeastern Brazil (Fig 1). Tremedal (14°58′21″S, 41°24′52″W) lies approximately 590 km from Salvador and has an estimated population of 15,996 inhabitants; it ranks among the lowest Brazilian municipalities for per capita income, with an economy based on subsistence agriculture in a semi-arid caatinga setting, and delivers primary care through three urban and seven rural health units. Novo Horizonte (12°48′28″S, 42°10′04″W), has an estimated population of 12,522 inhabitants and an economy based on rutile quartz extraction and garlic cultivation, and delivers primary care through two urban and four rural health units.

### Study design and data sources

This retrospective observational study evaluated records of individuals from Tremedal and Novo Horizonte for whom serological testing for Chagas disease was requested within the public primary healthcare network between 2019 and 2023. Requests originated from 14 health units, including five urban and nine rural facilities. Following the diagnostic algorithm recommended by the Brazilian Clinical Protocol and Therapeutic Guidelines (PCDT) [16], confirmatory serological testing for *T. cruzi* infection was requested through the Central Public Health Laboratory of Bahia (LACEN-BA). Tests were submitted by primary care physicians, mainly family physicians working in local family health strategy teams. At the time of this study, no systematic population-based serological screening program for CD had been implemented in Brazilian primary care. Testing was therefore requested at the discretion of individual clinicians, either because of clinical suspicion or during routine primary care encounters in which CD testing was considered appropriate.

Serological test and result data were retrieved from the Laboratory Environment Manager (GAL) system. According to the routine diagnostic workflow of this network, cases were considered confirmed when two reactive serological results were obtained using tests based on different antigenic principles: one based on recombinant antigens (BIOLISA Chagas Recombinante or Anti Chagas SYM, Vyttra Diagnósticos) and one based on electrochemiluminescence immunoassay (Elecsys Chagas, Roche). Confirmed cases were subsequently included for clinical assessment. Because the study was based on retrospective extraction of routine health system records, the specific clinical indications for serological testing were not consistently available. Therefore, it was not possible to determine whether individuals were tested due to symptoms, clinical suspicion, or asymptomatic screening. Clinical information was extracted from the Citizen Electronic Health Record module of e-SUS Primary Care (e-SUS APS/PEC), the electronic medical record system used in Brazilian primary care. The study evaluated demographic characteristics, clinical history, comorbidities, antiparasitic treatment indications, treatment use, and reported adverse drug reactions. Information related to diagnostic tests and procedures performed outside the study municipalities, including specialized consultations and surgical interventions carried out in referral centers, was unavailable when these data were not documented in the local e-SUS APS/PEC record.

### Clinical assessment and classification

The analysis focused on individuals with confirmed chronic CD. Available clinical records were reviewed to identify cardiac and digestive manifestations. Complementary test results were assessed when available, including electrocardiograms (ECG), echocardiograms (ECHO), chest radiography, colonoscopy, and laboratory findings. Cardiac involvement was evaluated based on reported symptoms, ECG abnormalities, and ECHO findings. Digestive involvement was defined according to clinical symptoms, imaging studies, endoscopic findings, or previous surgical history compatible with CD. Clinical forms were classified as indeterminate, cardiac, digestive, or mixed. ECG and ECHO findings were categorized as typical of chronic Chagas cardiomyopathy or nonspecific, according to the Brazilian Society of Cardiology Guideline on the Diagnosis and Treatment of Patients with Chagas Cardiomyopathy [17], Biolo et al. [18], and Hasslocher-Moreno et al. [19]. The cardiac form was assigned when at least one typical alteration was documented; the digestive form when megaesophagus or megacolon was confirmed by imaging, endoscopy, or surgery; the mixed form when definitive digestive involvement coexisted with any documented cardiac alteration; and the indeterminate form in seropositive individuals with normal or only nonspecific findings and no definitive digestive disease. Information on antiparasitic treatment with benznidazole included prescription records, reported contraindications, and adverse events when documented in the PEC system.

### Turnaround time assessment

Diagnostic efficiency was assessed through turnaround time (TAT), defined as the interval between blood sample collection and availability of serological results in the GAL system. TAT values were calculated for individuals with complete date information and analyzed separately for each municipality.

### Data analysis

Statistical analyses were performed using MedCalc software (version 23.4.5). Continuous variables were summarized as medians and interquartile ranges (IQR), whereas categorical variables were expressed as absolute frequencies and percentages. Given the descriptive and exploratory nature of the study, no inferential statistical tests were performed.

## Results

### Serological testing and temporal trends

Between 2019 and 2023, a total of 472 individuals underwent serological testing for CD in the municipalities of Tremedal and Novo Horizonte. In Tremedal, test requests increased markedly over time, rising from 25 in 2019–229 in 2023, representing an almost tenfold increase over the study period. In contrast, Novo Horizonte initiated serological testing only in 2023, with 13 recorded tests requested.

### Demographic characteristics of tested individuals

Among the 459 serological tests submitted in Tremedal, 343 (74.7%) were women and 105 (22.9%) were men, resulting in a female-to-male ratio of 3.3:1. Sex information was unavailable in 11 cases (2.4%). Age data were available for 400 individuals, with a median age of 32 years (IQR: 24–45.8). Men presented a significantly higher median age than women (47 years (IQR: 33–61) vs 29 years (IQR: 23–39), respectively). Self-reported race/ethnicity in Tremedal identified 71 individuals (15.5%) as white, 81 (17.6%) as mixed-color, and 14 (3.1%) as black; race data were missing for 293 individuals (63.8%).

In Novo Horizonte, 10 serological tests (76.9%) were women and 3 (23.1%) were men. Overall, five individuals self-identified as white (38.5%), seven as mixed-color (53.8%), and one did not report race/ethnicity (7.7%). The overall median age was 30 years (IQR: 24.5–43), with no significant difference between men (32 years, IQR: 25–50) and women (29.5 years, IQR: 22.3–40).

### Distribution of test requests by healthcare unit and professional engagement

In Tremedal, most serological tests originated from urban primary healthcare units (221; 55.3%). Rural units accounted for 178 tests (44.7%), primarily from São Felipe (n = 58; 32.4%), Furado da Cancela (n = 50; 27.9%), Lagoa Preta (n = 42; 23.5%), Venda Velha (n = 19; 10.6%) and São João dos Britos (n = 10; 5.6%). A total of 43 healthcare professionals submitted serological tests in Tremedal. However, three physicians accounted for 179 of the 459 tests (39%), indicating that a substantial proportion of testing was concentrated among a small group of clinicians. In Novo Horizonte, all tests originated from a single urban healthcare unit and were submitted by one professional.

### Turnaround time for serological testing

Turnaround time data were available for 404 tests in Tremedal. The median TAT was 14 days (IQR: 10–30). In Novo Horizonte, the median TAT was significantly lower at 10 days (IQR: 5.5–12.5 days), although this estimate derived from a smaller number of observations.

### Seropositivity and confirmed cases

Among the 472 serological tests performed, 14 individuals (3.0%) tested positive for CD, all from Tremedal. These cases originated from two healthcare units: São Felipe and Manoel Inácio Pereira. One case was excluded from further analysis due to incomplete clinical information, yielding a final sample of 13 confirmed chronic CD cases (Table 1). The median age of confirmed cases was 52 years (IQR 49–67.5), with a predominance of female individuals (61.5%; n = 8).

**Table 1:** Demographic characteristics of confirmed CD cases.

Variables	n (%) (N = 13)
**Sex**	
Female	8 (61.5%)
Male	5 (38.5%)
Female to male ratio	1.6:1
**Age (years)**	
Median (IQR)	52 (49 – 67.5)
10–19	1 (7.7%)
20–39	0 (0%)
40–49	4 (30.8%)
50–59	4 (30.8%)
60–69	2 (15.4%)
70–79	2 (15.4%)

### Clinical evaluation and disease classification

All 13 individuals underwent ECG assessment. Transthoracic ECHO reports were available for 10 of the 13 individuals; ECHO was not documented in the electronic medical records for remaining three (Table 2). When performed, ECHO was conducted without contrast agents. One individual underwent colonoscopy, and chest radiography results were unavailable for all cases. ECG abnormalities were identified in eight individuals and included right bundle branch block (RBBB), sinus bradycardia, QRS axis deviation, prolonged QT interval, second-degree atrioventricular block, ectopic rhythm, and ventricular repolarization changes. ECHO abnormalities were identified in one individual and consisted of mild aortic, mitral, and tricuspid valve insufficiency occurring concurrently. Other ECHO findings typically associated with Chagas cardiomyopathy, such as apical aneurysm, regional wall motion abnormalities, intracardiac thrombus, or diastolic dysfunction, were not reported.

**Table 2:** Narrative clinical profiles of chronic Chagas disease cases from Tremedal, Bahia, Brazil.

Sex	Age band*	Comorbidities	ECG findings	ECHO findings	Digestive involvement
**Cardiac form (n = 2)**					
M	40–49	Arterial hypertension	Second-degree AV block; RBBB with left anterior fascicular block; QT prolongation	Not performed	None
F	50–59	Arterial hypertension	Primary ventricular repolarization changes; left bundle branch conduction abnormality	Normal	None
**Mixed form (n = 2)**					
F	60–69	Arterial hypertension	RBBB	Mild aortic, mitral, and tricuspid valve insufficiency	Chagasic megacolon
F^†^	50–59	Arterial hypertension	RBBB; sinus bradycardia; QT prolongation; QRS axis deviation	Normal	Megaesophagus
**Digestive form (n = 1)**					
F	70–79	None	Normal	Normal	Megaesophagus
**Indeterminate form (n = 8)**					
F	40–49	Arterial hypertension	Normal	Normal	None
M	40–49	Arterial hypertension	Normal	Not performed (cardiac form not excluded)	None
M	70–79	None	Normal	Normal	None
F	10–19	None	Isolated RBBB	Normal	None
F	40–49	None	Normal	Normal	None
M	60–69	Arterial hypertension	Left atrial enlargement; QT prolongation; left anterior hemiblock	Normal	None
F	50–59	Arterial hypertension	Ventricular repolarization abnormality	Normal	None
M^†^	50–59	None	Ectopic rhythm	Not performed (cardiac form not excluded)	None

*Age in years.

†Patient received antiparasitic treatment with benznidazole (5–7 mg/kg/day, orally in two divided doses) for 60 days. Cases are grouped by clinical form. In the ECG and ECHO columns, “Normal” indicates that the examination was performed and showed no abnormalities, whereas “Not performed” indicates that no report was available in the electronic medical records. In two individuals classified with the indeterminate form, ECHO was not available and subclinical cardiac involvement could not be fully excluded. The classification of ECG and ECHO findings as typical or nonspecific for chronic Chagas cardiomyopathy is presented the Discussion, following the criteria of Biolo et al. [18] and the Brazilian Society of Cardiology Guideline on Chagas Cardiomyopathy [17]. Abbreviations: AV: Atrioventricular; ECG: Electrocardiogram; ECHO: Echocardiogram; RBBB: Right bundle branch block.

Clinical classification identified the indeterminate form in eight individuals (61.5%), the cardiac form in two (15.4%), the mixed form in two (15.4%), and the digestive form in one (7.7%). Among the eight individuals classified with the indeterminate form, two had a normal or nonspecific ECG without an available ECHO report; in these cases, subclinical cardiac involvement could not be entirely excluded, and they were retained in the indeterminate group with this caveat (Table 2). Application of standardized ECG criteria for chronic Chagas cardiomyopathy reclassified several conduction and repolarization findings as nonspecific rather than typical of Chagas cardiomyopathy, supporting the predominance of the indeterminate form.

### Digestive manifestations and outcomes

Digestive involvement was documented in three individuals. One individual underwent Duhamel surgery for Chagasic megacolon. A second individual was a woman aged 50–59 woman with a history of surgery for megaesophagus and persistent dyspepsia. Another individual, aged 70–79, presented with megaesophagus secondary to achalasia. One individual, aged 70–79, died during the study period; the cause of death could not be determined due to limited information.

### Antiparasitic treatment

All confirmed cases received referral for clinical follow-up and assessment for antiparasitic therapy. Benznidazole was prescribed in two individuals with no documented contraindications. The electronic medical records did not report adverse drug reactions in these cases.

## Discussion

This retrospective analysis reveals marked disparities in the detection and clinical management of Chagas disease between the municipalities of Tremedal and Novo Horizonte, Bahia, during the period preceding the full implementation of the Oxente Chagas Bahia Project [15]. Although both municipalities are historically endemic, serological testing expanded substantially only in Tremedal, whereas it remained minimal in Novo Horizonte until 2023. These findings illustrate how local health system organization, professional engagement, and programmatic prioritization strongly influence access to diagnosis in endemic settings.

Among the 13 confirmed cases analyzed, the clinical spectrum was broad, encompassing cardiac, digestive, mixed and indeterminate forms, consistent with long-standing infection and delayed diagnosis. Importantly, the identification of a pediatric case (aged 10–19 years) with confirmed CD raises concerns for ongoing transmission, either through residual vectorial activity or congenital infection, and highlights persistent surveillance gaps that extend beyond the adult population. Because maternal infection status was not available in the records, congenital transmission could not be assessed.

In Tremedal, the concentration of 39.0% of serological tests among three family physicians suggests that Chagas disease screening was largely driven by a small group of particularly engaged clinicians, consistent with the concept of local “Chagas champions” within primary care. These physicians worked within family health strategy teams and had no additional specialization, indicating that their contribution likely reflected individual clinical awareness and local leadership rather than specialist-driven care. Their heightened engagement in CD screening predated the formal activities of the Oxente Chagas Bahia Project, which began influencing testing practices in Tremedal only from 2022 onward. In contrast, testing in Novo Horizonte relied on a single professional from one urban health unit, despite both national guidelines [16] and a 2022 joint technical note from the Bahia State Health Secretariat explicitly recommending routine Chagas disease screening, including during prenatal care, in endemic municipalities [20]. This marked asymmetry suggests inconsistency implementation of guideline-directed care across the primary healthcare network and underscores the importance of continuous professional training, locally empowered clinical leadership, and individual accountability within primary care teams to ensure effective Cd detection in endemic regions.

Turnaround time for serological testing also differed between municipalities, with a median of 14 days in Tremedal and 10 days in Novo Horizonte. This difference likely reflects variations in laboratory processing capacity, transport logistics, and testing frequency. Samples from Novo Horizonte are processed at the Central Public Health Laboratory of Bahia (LACEN-BA) in Salvador, whereas samples from Tremedal are sent to a regional LACEN branch in Vitória da Conquista, which operates with lower testing volumes and does not perform daily analyses. Although no direct clinical consequences of delayed results were documented, prolonged turnaround times may discourage test requests, delay confirmatory diagnosis, hinder timely clinical staging, and make it more difficult to contact individuals after testing, particularly in rural settings with limited follow-up infrastructure.

Clinical characterization of confirmed cases revealed a predominance of the indeterminate form, consistent with reports from highly endemic rural areas, where the indeterminate form is the most frequent clinical presentation of chronic CD [21,22]. Among individuals with cardiac involvement, ECG abnormalities such as second-degree atrioventricular block, left anterior fascicular block, bradyarrhythmia, and ventricular repolarization changes represent classical findings associated with Chagas cardiomyopathy [23]. In the present series, second-degree atrioventricular block, right bundle branch block associated with left anterior fascicular block, and primary ventricular repolarization changes were regarded as typical of chronic Chagas cardiomyopathy, whereas isolated right bundle branch block, sinus bradycardia, QT prolongation, QRS axis deviation, left atrial enlargement, and ectopic rhythm were considered nonspecific. Specific ECG abnormalities, including atrial fibrillation, electrically inactive areas, and non-sustained ventricular tachycardia, carry adverse prognostic implications, as they have been associated with ventricular dysfunction, heart failure, and sudden cardiac death in chronic Chagas cardiomyopathy [17]. Valvular insufficiency, reported in one seropositive individual, is not a typical manifestation of Chagas cardiomyopathy; in the absence of left ventricular ejection fraction data, it cannot be attributed to Chagas-related myocardial dysfunction. The cardiac manifestations observed are consistent with patterns described in both Brazilian [24,25] and international cohorts [26]. The relative frequency of digestive forms, by contrast, varies across geographic and epidemiological contexts [27].

In the present cohort, digestive involvement was notably frequent. Three of the 13 confirmed cases (23.1%) presented with megaesophagus or megacolon, a proportion higher than the 10–15% typically reported in endemic cohorts; megavisceral disease, however, spans a spectrum of severity, and not all affected individuals progress to advanced or surgically managed forms [27–29]. One individual underwent Duhamel surgery for Chagasic megacolon, while two others presented with advanced esophageal disease, including megaesophagus with a history of surgical intervention and persistent dyspepsia, and megaesophagus secondary to achalasia. These conditions carry profound consequences for patients’ daily lives. Progressive dysphagia and chronic constipation impair nutritional intake and restrict physical capacity, often limiting individuals’ ability to perform occupational tasks and sustain income-generating activities, a particularly significant burden in the rural, low-income communities where these patients reside. The need for surgical intervention further compounds this impact through prolonged recovery, healthcare costs, and potential loss of productive capacity.

Despite the identification of individuals eligible for etiological treatment according to the Brazilian Clinical Protocol and Therapeutic Guidelines [16], documentation of benznidazole use was limited to two cases. Both treated individuals were older than 50 years. One presented a mixed cardiac–digestive form with established cardiac involvement; the other had only an unspecified arrhythmia and was classified as the indeterminate form. The treatment of an individual with established cardiac involvement is noteworthy in light of the BENEFIT trial, which demonstrated that benznidazole did not significantly reduce cardiac clinical deterioration in patients with established Chagas cardiomyopathy [30], suggesting that the individuals treated in this cohort may not have been the optimal candidates for etiological therapy. In addition, the Brazilian PCDT explicitly states that etiological treatment is not recommended as routine practice for individuals over 50 years of age, given the greater uncertainty of benefit in this group [16]. The absence of documented adverse drug reactions in these two individuals is noteworthy; however, given the retrospective nature of the study and the limitations of routine clinical records, this finding cannot be interpreted as evidence of tolerability. Conversely, several younger individuals, who would be more likely to benefit from treatment in terms of parasitological response and prevention of disease progression, did not receive benznidazole. Notably, the pediatric patient (aged 10–19 years)had no documented treatment, and available records suggest that the family relocated from the municipality before treatment could be initiated, precluding follow-up and care. These findings highlight an important gap in guideline-concordant care and have direct implications for physician education and the design of future screening interventions in endemic settings.

Detection of CD in a child further emphasizes the urgency of strengthening integrated surveillance strategies. Pediatric infection indicates either recent transmission or failure to identify congenital cases. Despite advances in vector control, the continued capture of native triatomine species, including *T. cruzi*-infected specimens, in both municipalities suggests that vectorial transmission risk has not been fully eliminated [13,14]. However, as congenital transmission could not be excluded in this case, the source of infection in the pediatric patient remains uncertain. Intensive vector surveillance, systematic household investigation, maternal screening, and community engagement remain essential to prevent new infections and interrupt transmission cycles [31,32].

The age distribution of confirmed cases in this cohort may partly reflect the impact of vector control programs in the region, but it should also be interpreted in light of healthcare-seeking and testing bias. The absence of confirmed cases in the 20–39-year age group is consistent with the progressive reduction in domiciliary triatomine burden that occurred during the formative years of individuals now in this age range, as intensified control efforts preceded the last detection of *Triatoma infestans* in Tremedal and Novo Horizonte in 2011 and 2015, respectively. However, adults aged 20–39 years may also be underrepresented in routine primary care-based testing because they are often generally healthy, economically active, and less likely to seek medical care, except in contexts such as prenatal care. Therefore, the absence of confirmed cases in this age group should not be interpreted as definitive evidence of absence of infection. In contrast, the predominance of confirmed cases among individuals aged 40 years and older likely reflects cumulative exposure acquired prior to the consolidation of vector control. Together, these observations underscore the importance of maintaining active vector surveillance and systematic screening across all age groups in endemic settings.

The case of an individual aged 70–79 whose cause of death could not be determined illustrates broader structural deficiencies in rural healthcare systems, including limited diagnostic capacity, poor integration of clinical information, and restricted access to specialized care, all of which compromise comprehensive management of Chagas disease in endemic settings. Early diagnosis and etiological treatment remain critical to prevent progression to advanced chronic forms of CD, in which symptoms become more severe, cumulative, and less predictable. Rapid diagnostic tests represent essential tools to expand early screening and improve timely access to care.

This study presents limitations inherent to its retrospective design. Because testing was initiated at the discretion of individual clinicians rather than through systematic population-based screening, the 3.0% seropositivity rate reflects outcomes among those who sought or were offered testing and should not be interpreted as a prevalence estimate. In addition, diagnostic procedures and specialist evaluations performed outside the municipalities frequently lacked documentation in local electronic health systems. This limitation may have led to an underestimate of disease severity, as complications and specialist findings from referral centers were not necessarily captured. Conversely, management gaps may have been overestimated, as some referrals, follow-up, or treatment may have occurred outside the local public primary care records and therefore remained undocumented. Clinical classification was further constrained by the incomplete availability of echocardiographic data: ECHO reports were not documented for three of the 13 individuals, so subclinical cardiac involvement could not be fully excluded in the two individuals with a normal or nonspecific ECG who were classified with the indeterminate form. Moreover, the ECHO examinations that were available had been performed without contrast, which reduces sensitivity for detecting abnormalities specifically associated with Chagas cardiomyopathy, such as apical aneurysm and intracardiac thrombus. Nonetheless, these limitations reflect real-world structural deficiencies in rural health networks.

This analysis documented a persistent burden of chronic CD in Tremedal prior to the implementation of the Oxente Chagas Bahia Project, with a predominance of the indeterminate form alongside cardiac and severe digestive manifestations and critical gaps in treatment uptake. In Novo Horizonte, the absence of confirmed cases most likely reflects limited screening rather than true absence of disease. The identification of a pediatric case (aged 10–19 years), together with the age distribution of confirmed cases, suggests that transmission may be ongoing and reinforces the need for active vector surveillance and screening across all age groups. These findings establish a real-world baseline for evaluating the impact of the Oxente Chagas Bahia Project and identify actionable gaps in screening, diagnosis, and guideline-concordant management in endemic settings.
